# Biochar Production from Sugarcane Residual Biomass Lignin Through Pyrolysis

**DOI:** 10.3390/ma18235321

**Published:** 2025-11-26

**Authors:** Thiago Claudino Mendes de Almeida, Hélio Merá de Assis, Sarah Inglid dos Santos Silva, Angela Maria Tribuzy de Magalhães Cordeiro, Ramon Kenned Sousa Almeida, Nataly Albuquerque Dos Santos

**Affiliations:** 1 Graduate Program in Chemistry, Center for Exact and Natural Sciences, Department of Chemistry, Federal University of Paraíba, João Pessoa 58051-900, PB, Brazil; sarahidnts@gmail.com; 2 Senai Institute for Innovation in Biomass, Três Lagoas 79640-250, MS, Brazil; hassis@ms.senai.br; 3 Department of Food Technology, Center for Technology and Regional Development, Federal University of Paraíba, João Pessoa 58051-900, PB, Brazil; atribuzycordeiro@gmail.com; 4 Serra Talhada Academic Unit, Federal Rural University of Pernambuco, Serra Talhada 56909-535, PE, Brazil; ramon.almeida@ufrpe.br

**Keywords:** sugarcane, lignin, biochar, pyrolysis

## Abstract

Lignin is the most structurally complex component of lignocellulosic biomass. Each year, thousands of tons of lignin-rich residues from enzymatic hydrolysis are generated in sugarcane-based cellulosic ethanol biorefineries. The current study specifically utilizes lignin extracted from sugarcane bagasse as the primary feedstock for biochar production, rather than employing the raw bagasse itself. This study investigates, through pyrolytic thermal treatment of two lignin sources, the production of biochars and the evaluation of their potential applications. Kraft commercial lignin and sugarcane bagasse lignin samples, along with their corresponding biochars, were characterized by elemental and proximate analyses, higher heating value determination, spectroscopic techniques, thermogravimetric analysis, X-Ray diffraction, scanning electron microscopy coupled with energy-dispersive spectroscopy, and true density measurements. The results revealed a lower contamination level associated with the extraction process and confirmed the amorphous nature of sugarcane bagasse lignin and its derived biochar. An O/C ratio of approximately 0.3 was obtained for the sugarcane bagasse lignin biochar based on both elemental and Raman spectroscopy analyses. Both elemental composition assessment and Raman spectroscopic analysis indicated that all biochar specimens exhibited hydrogen-to-carbon (H/C) ratios exceeding 0.5. The analyses, therefore, indicated that the biochar derived from sugarcane lignin exhibited higher energy density, moderate stability and a high carbon content. The proposed approach thus provides promising alternatives for the valorizing lignin residues derived from second-generation ethanol production processes.

## 1. Introduction

Brazil stands as one of the global leaders in second-generation ethanol (E2G) production. This alternative energy source is obtained through the biological fermentation of sugars derived from lignocellulosic biomass. Although sugarcane bagasse and straw are the main feedstocks used in second-generation ethanol biorefineries, other agricultural residues—such as corn stover, agave bagasse, wood, and cellulosic paper waste—can also serve as potential raw materials [1].

The continuous advancement of E2G production technologies, particularly those based on sugarcane bagasse, has led to the generation of large quantities of residual materials [2].

Sugarcane bagasse (*Saccharum officinarum*) typically consists of approximately 32.7% to 46.4% cellulose, 20.2% to 29.6% hemicellulose, 18.9% to 27.5% total lignin, and 3.1% to 10.3% extractives [3]. In 2024, according to the Brazilian Institute of Geography and Statistics (IBGE), sugarcane production in Brazil reached 759.662.482 metric tons. During its processing, various residues and by-products are generated, including straw, bagasse, boiler ash, and vinasse. Bagasse is primarily utilized in boilers or cogeneration units to supply heat and power [4].

Certain pretreatment and processing technologies can disrupt the fibrous structure of bagasse, enabling access to cellulose and hemicellulose sugars required for ethanol synthesis. During steam explosion, a reactor is operated under elevated pressures and temperatures to carry out the pretreatment of sugarcane bagasse using water vapor. Following the disruption of the bagasse structure, the material can be treated with a sodium hydroxide solution, which is capable of solubilizing lignin and a portion of the hemicellulose. Subsequently, lignin can be recovered from the resulting black liquor.

Despite the entirety of this process, most of the lignin generated during E2G production is still discarded or combusted for bioenergy generation [5].

Globally, it is estimated that less than 2% of the lignin generated by the pulp and paper industry is employed in the manufacture of high-value products such as dispersants, adhesives, and surfactants [5]. In turn, in a typical sugarcane-based cellulosic ethanol biorefinery producing 80 million liters annually, approximately 70–130 thousand tons of lignin-rich enzymatic hydrolysis residue are generated annually [6].

Alternative extraction methods, such as organosolv and alkaline processes, still face limitations in producing lignin with low degradation, high purity, and satisfactory yields [7,8].

Kraft lignin holds a larger market share compared to soda lignin, lignosulfonates, and organosolv lignin, with an estimated cost ranging from USD 250 to USD 364 per ton. During the cooking process in the production of kraft lignin, the lignin present in the wood is solubilized in an aqueous solution of sodium hydroxide and sodium sulfide. Subsequently, lignin can be extracted from the black liquor. The kraft alkaline extraction method, commonly employed in the paper industry, produces lignin with a higher sulfur content. Its application is already well-established at an industrial scale, making it an appropriate benchmark for comparison with lignins derived from other biomass sources using different extraction methods [9].

Due to its highly thermoresistant and carbonaceous nature, lignin can be effectively modified only through thermochemical processes. Among these, pyrolysis—thermal decomposition in the absence of oxygen—produces various industrially relevant products. In particular, slow pyrolysis favors the formation of a solid carbonaceous material known as biochar.

Biochar is recognized by the United Nations Intergovernmental Panel on Climate Change as both a carbon dioxide removal (CDR) technology and a viable market-based solution. The CDR potential of biochar derives from the CO_2_ initially captured via photosynthesis during biomass growth, which is subsequently converted into a stable form of carbon through pyrolysis [10,11].

Despite the growing interest in lignin valorization, few studies have focused on the use of lignin residues from sugarcane bagasse–based E2G production for biochar synthesis. The valorization of lignocellulosic biomass into value-added products through hydrothermal treatment (<300 °C) has been explored [12]. A previous study [6] investigated the production of bio-oil and biochar from lignin-rich residues derived from sugarcane bagasse. However, the present study specifically employs lignin isolated from sugarcane bagasse as the feedstock for biochar production, rather than raw bagasse itself. This approach enhances the valorization of a more recalcitrant and underutilized by-product.

In this context, lignin-derived biochar may provide enhanced energy efficiency when utilized as a solid fuel. Additionally, the valorization of lignin residues involves their transformation into materials capable of long-term carbon storage [13].

Biochar is composed primarily of carbon, oxygen, and heteroatoms, although mineral inclusions are frequently present and may limit its range of applications. Its composition is highly dependent on the precursor material and influenced by pyrolysis conditions.

In this study, slow pyrolysis of kraft lignin and sugarcane bagasse lignin was conducted with the objective of maximizing the yield of the solid fraction.

Ref. [14] indicate that conventional slow pyrolysis processes typically operate within a temperature range of 300 to 700 °C. Therefore, the selection of 500 °C as the pyrolysis temperature is supported by existing literature. Within this temperature range, an intermediate value may maximize the carbon yield of the resulting biochar relative to the original lignin, minimize potential losses in biochar yield, while concurrently enhancing the efficiency of thermal energy utilization during the pyrolysis process.

Many studies have reported biochars suitable for soil enhancement, carbon sequestration, environmental remediation, and as precursors for carbon-based materials. Moreover, biochars can improve the mechanical, thermal, and electrical properties of polymer composites and offer additional technological applications [15,16,17,18,19,20,21,22].

In light of these considerations, the potential applications of the resulting biochars were evaluated based on their suitability as solid fuels (assessed via energy density) and as stable carbon storage materials (assessed via stability).

The pyrolysis conditions were designed to limit the removal of surface functional groups and to assess the effect of this approach on the higher heating value (HHV) of the resulting biochars, given that elevated oxygen-to-carbon (O/C) ratios—although associated with increased cation exchange capacity (CEC)—tend to reduce the calorific value of biochar [23].

Beyond energetic applications, the moderate oxygen-to-carbon (O/C) ratios of lignin-derived biochars may contribute to other functions. A high O:C ratio is desirable must not be excessively high, as it would reduce the biochar’s half-life. The low (O/C) of biochars produced from conventional feedstocks limits their commercial applications; therefore, an intermediary O:C ratio in lignin-based biochars could offer an alternative outcome.

Furthermore, lignin-rich biomass can support decarbonization efforts and promote a circular economy, as lignin is the second most abundant renewable resource derived from lignocellulosic biomass.

## 2. Materials and Methods

### 2.1. Materials

Two types of lignin were used in this study: commercial kraft lignin (LK) (Sigma Aldrich Co., St. Louis, MO, USA), employed as a reference, and lignin extracted from sugarcane bagasse (LCA). The latter was obtained through a steam explosion pretreatment at 200 °C for 7 min in a 5 m^3^ reactor. Delignification was carried out using 1 wt% NaOH in a 350 L reactor at the Escola de Engenharia de Lorena (EEL-USP).

### 2.2. Thermal Analyses of Sugarcane Bagasse Lignin and Kraft Lignin

To determine the pyrolysis conditions of the sugarcane bagasse lignin, the effect of the different heating rate on the thermogravimetric curves was previously evaluated. Approximately 10 mg of sugarcane bagasse lignin were subjected to a non-isothermal heating program from room temperature to 1000 °C under a high-purity nitrogen atmosphere (99.997%) at a flow rate of 50 mL·min^−1^. Alumina crucibles were used in the analyses with thermal analyzer (Discovery SDT650, TA Instruments, Hüllhorst, Germany). Heating rates of 10 °C· min^−1^ (LCA10), 20 °C·min^−1^ (LCA20), and 30 °C·min^−1^ (LCA30) were evaluated.

Thermogravimetric analysis of kraft lignin (KL) was conducted under an inert atmosphere with a gas flow rate of 100 mL·min^−1^ and a heating rate of 10 °C.min^−1^. Alumina crucibles were used to analyze approximately 10 mg of sample in experiments performed on a thermobalance in a thermal analyzer (Discovery SDT650, TA Instruments, Hüllhorst, Germany), in which the sample were heated up to 850 °C.

### 2.3. In Situ Pyrolysis in a Fixed Bed Reactor

Pyrolysis experiments were performed in situ to produce solid, liquid, and gaseous fractions. Lignin samples, either from sugarcane bagasse or commercial kraft lignin, were pyrolyzed under controlled conditions.

For each experiment, 10 g of lignin were weighed on an analytical balance. The fixed-bed reactor was purged for 20 min with nitrogen gas before heating. The gaseous products were condensed, and the furnace temperature was controlled via a computer-based command system. All experiments were carried out in a horizontal tubular furnace (FT-1200/H model, Fortelab, São Paulo, Brazil).

The pyrolysis process was adapted based on the previous thermal analysis of sugarcane bagasse lignin, with the objective of increasing the yield of the solid fraction.

The selected temperature of 500 °C was also supported by the literature, as authors such as [14] report that typical conditions for slow pyrolysis involve temperatures ranging from 300 to 700 °C. Within this broad temperature range, an intermediate value was chosen to enhance the carbon content of the resulting biochar compared to the original lignin, while maintaining low thermal energy input, as reflected in the residence time and the final temperature employed during pyrolysis.

Optimization based on the heating rate variation indicated negligible standard deviations, with approximately 0% variation in kraft lignin biochar yield and about 2% variation for sugarcane bagasse lignin biochar yield.

The selected pyrolysis parameters were as follows: heating rate of 20 °C·min^−1^, nitrogen flow of approximately 100 mL·min^−1^, final pyrolysis temperature of 500 °C, and residence time at the final temperature of 15 min. The resulting biochars were labeled as K20 (kraft lignin biochar) and CA20 (sugarcane bagasse lignin biochar).

Biochar yield, adapted from [24], was calculated on a dry basis to eliminate moisture-related bias. The yield (mass %) was determined as the ratio between the mass of dried biochar obtained after pyrolysis and the mass of dried lignin feedstock.

### 2.4. Proximate Analysis and Determination of the Higher Heating Value

Proximate analyses of the biochars were carried out according to the methodology described by [25]. Thermogravimetric analyses were conducted under both nitrogen and synthetic air atmospheres (100 mL·min^−1^) at a heating rate of 10 °C·min^−1^, using alumina crucibles and 10 mg of each sample in a thermobalance (Discovery SDT650, TA Instruments, Hüllhorst, Germany), heating up to 600 °C.

Moisture content was defined as the mass loss between 30 °C and 105 °C under air. Ash content corresponded to the residual mass at 600 °C under air. Volatile matter was defined as the mass loss between 105 °C and 600 °C under both synthetic air (MVar) and nitrogen (MVN_2_). For accurate volatile determination, the nitrogen atmosphere curve was used since oxidation under air may affect fixed carbon estimation. The fixed carbon content (%) was obtained from the difference (MVar—MVN_2_).

The Higher Heating Value (HHV) was measured using an IKA c-200 calorimeter (IKA Group, IKA-Werke GmbH & Co. KG, Staufen, Germany), in triplicate, following ASTM D5865 [26].

### 2.5. Thermal Profile of Biochars

To assess the thermal behavior at higher temperatures, thermogravimetric analyses (TG) of the biochar samples were performed using a simultaneous thermal analyzer (STA 449 F3 JUPITER^®^, NETZSCH, NETZSCH-Gerätebau GmbH: Selb, Germany). Approximately 20 mg of each sample were placed in alumina crucibles and heated from 30 °C to 950 °C at a rate of 20 °C·min^−1^ under an inert nitrogen atmosphere (20 mL·min^−1^).

### 2.6. Elemental Analysis

Elemental analyses (C, H, N, S) of lignins and biochar samples were obtained using a Unicube^®^ Elemental Analyzer (Elementar Analysensysteme GmbH, Langenselbold, Germany). Approximately 1 mg of each sample was analyzed in duplicate. In this elemental analysis technique, the results are based on the entire product, as the mass includes the ash content.

The oxygen content of the biochars was determined using the following equation:

O(%) = 100 − C(%) − H(%) − N(%) − S(%) − Ash(%).

### 2.7. X-Ray Diffraction of Lignins and Biochars

X-Ray diffraction analysis was performed using a Shimadzu Lab X XRD-6000 diffractometer (Kyoto, Japan). Both lignin and biochar samples were analyzed using CuKα radiation (λ = 1.5406 Å), at 30 kV and 30 mA, under ambient temperature. Scans were conducted at a rate of 2° min^−1^ over a 2θ range of 2° to 55°.

### 2.8. Fourier Transform Infrared Spectroscopy (FTIR)

Infrared spectra of the *in natura* lignin and biochar samples were acquired using a Nicolet iS10 spectrometer (Thermo Scientific, Thermo Fisher Scientific/Nicolet—Madison, WI, USA), employing the Attenuated Total Reflectance (ATR) sampling technique with a diamond crystal. The samples were oven-dried at 60 °C for at least 1 h 30 min and subsequently stored in a desiccator. Analyses were carried out in duplicate, with 32 scans per measurement, in the spectral range of 4000–400 cm^−1^, using transmittance (%T) mode and a spectral resolution of 4 cm^−1^.

### 2.9. Raman Spectroscopy

Raman spectra were collected using a Renishaw InVia confocal Micro-Raman (Renishaw, Wotton-under-Edge, UK) using an Ar laser (514 nm) with the output power set at 2.5 mW power. The spectral range analyzed was 100–3700 cm^−1^ and the equipment was operating with a detector exposure time of 10 s and 10 accumulations per sample.

The D and G bands were fitted using Gaussian curve fitting, with coefficients of determination (R^2^) of 0.98483 for kraft lignin biochar and 0.99149 for sugarcane bagasse lignin biochar.

Intensities at 800 cm^−1^ (IAr), 1800 cm^−1^ (IBr), and the D band intensity (IDr)were used to calculate several derived parameters, following the equations proposed by [27]. Several references used these calculations, which are already consolidated in the area of carbonaceous materials [28,29,30,31].

These were applied to calculate parameters including the fluorescence interference coefficient (α), biochar yield (CY), residual volatile content (RV), volatile matter (Vdaf), and the H/C and O/C ratios:

α = (IBr − IAr)/(IDr − IAr)(1)

CY = 26.53 + 30.1758 ∗ α(2)

RV = −23.70 + 1.07735 ∗ CY(3)

Vdaf = −23.70/CY + 1.07735(4)

H/C = −0.0144+ 0.01769 ∗ Vdaf(5)

O/C = 0.0077 + 0.3253 ∗ (H/C)+ 0.0647 ∗ (H/C)^2^ (6)

### 2.10. Scanning Electron Microscopy Coupled with Energy Dispersive Spectroscopy

Samples were dehydrated, mounted on aluminum stubs with carbon tape, and sputter-coated with a ~2 nm gold layer using a Quorum Q150T E coater (Quorum, San Jose, CA, USA) under argon (20 mA, 120 s).

Surface morphology was observed using a Zeiss EVO LS15 high-resolution scanning electron microscope (Carl Zeiss Microscopy, Cambridge, UK) operated under high vacuum at up to 20 kV in backscattered electron (BSE) mode. Magnifications varied between 300× and 15.000×.

Elemental composition was determined using an Oxford Instruments (Oxford, UK) INCA X-act EDX detector (133 eV resolution for Mn Kα) operated at 20 kV. Analyses were performed on representative regions (30–60 s acquisition per point or map). Data were processed using Aztec 6.2 software with ZAF corrections to quantify major and trace elements (Fe, Si, Ca, K, Mg, Al).

### 2.11. True Density Analysis

True densities of kraft lignin, sugarcane bagasse lignin, and their respective biochars were determined after pre-drying, cooling, and grinding the materials.

This technique is widely employed for the characterization of solid materials such as lignins, biochars and other biomass-derived products, providing critical information about their internal structure and degree of compactness. In this procedure, the samples were initially oven-dried to a constant mass, cooled in a desiccator, and ground to obtain homogeneous particles. They were subsequently weighed with precision and analyzed using a helium gas pycnometer (Micromeritics AccuPyc 1330, Micromeritics, Norcross, GA, USA), an instrument that determines the true volume of the sample by measuring the pressure variation between chambers of known volume. The true density was calculated as the ratio of the dry mass to the measured volume. The average of five measurements was used to ensure repeatability.

## 3. Results and Discussion

### 3.1. Lignin

**Figure 1:**
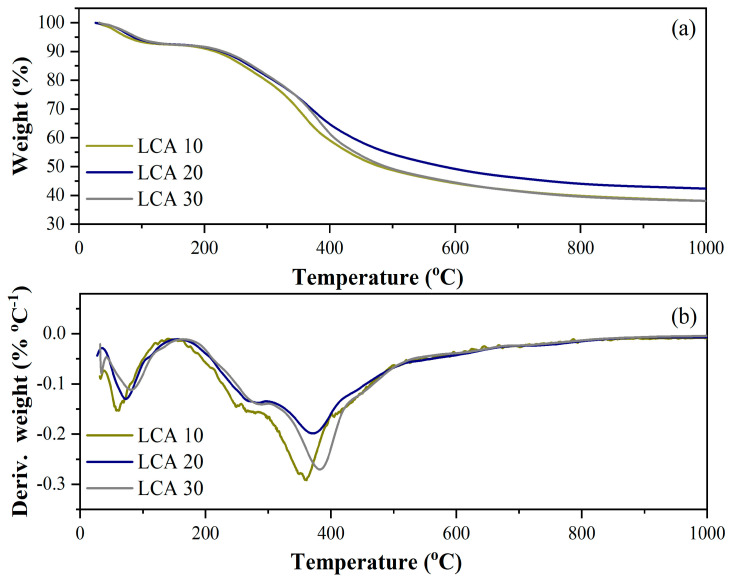
Thermal profile of sugarcane bagasse lignin under a nitrogen atmosphere at heating rates of 10 °C min^−1^, 20 °C min^−1^, and 30 °C min^−1^. Thermogravimetric curves (**a**) and derivative mass-loss curves (**b**).

The thermal decomposition profiles of sugarcane bagasse lignin at different heating rates are presented in Figure 1. The decomposition behavior of sugarcane bagasse lignin exhibited an initial mass-loss stage associated with dehydration, occurring up to approximately 150 °C, where a weight reduction of about 8% was observed for the samples heated at 10 °C·min^−1^ (LCA10), 20 °C·min^−1^ (LCA20), and 30 °C·min^−1^ (LCA30).

The second stage of decomposition—evidenced by a shoulder in the derivative thermogravimetric (DTG) curves—was observed between 250 °C and 290 °C and can be attributed to the degradation of residual hemicellulose and cellulose. Beyond this range, up to 1000 °C, the major decomposition event corresponded to the breakdown of the lignin aromatic structure.

The DTG curves indicated maximum decomposition rate temperatures, or peak temperatures (Tmax) ranging from 360 °C to 385 °C. Among the three thermal conditions tested, the highest Tmax occurred at the highest heating rate, while the lowest was observed at the lowest rate. This shift in the DTG peak toward higher temperatures with increasing heating rate is consistent with the low thermal conductivity of lignin and the presence of thermal lag effects [32].

At 1000 °C, the residual mass ranged from approximately 38% to 42%. The highest residual mass was obtained at a heating rate of 20 °C min^−1^, which was therefore selected for subsequent pyrolysis experiments to maximize the yield of the solid biochar fraction.

**Figure 2:**
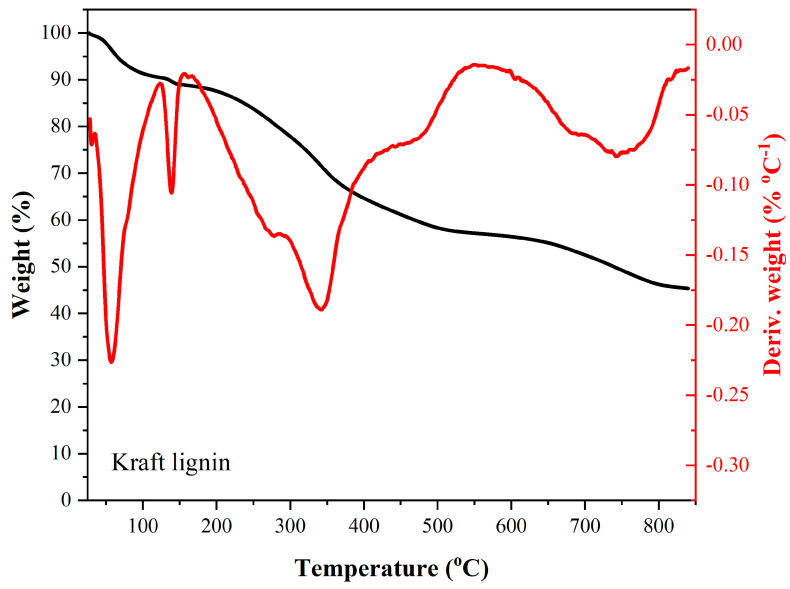
Thermal profile of kraft lignin under a nitrogen atmosphere.

Among the main stages of mass loss of kraft lignin under a nitrogen atmosphere—analyzed from the thermogravimetric (TG) and derivative thermogravimetric (DTG) curves shown in Figure 2—a mass loss of nearly 10% is observed up to approximately 120 °C, which can be attributed to the evaporation of moisture from the sample. Between 120 °C and 170 °C, a second region of mass loss of about 2% is identified, also associated with the dehydration of kraft lignin. Subsequently, a shoulder appears in the DTG curve, primarily related to the degradation of residual sugars, occurring between approximately 170 °C and 295 °C, resulting in a 10% mass loss. Another significant mass loss region, mainly attributed to the degradation of sugars and lignin, is observed between 295 °C and 420 °C, with a maximum degradation rate (Tmax) around 345 °C. Beyond 420 °C, a final stage of mass loss occurs, with a Tmax near 750 °C, corresponding to further thermal degradation of the lignin structure. At the final analysis temperature of approximately 850 °C, a residual mass of about 46% remained.

### 3.2. Biochar Production, Proximate Analysis, and Higher Heating Value

The yield of biochar derived from sugarcane bagasse lignin (53.20%) was consistent with values previously reported in the literature [33]. In contrast, the biochar produced from kraft lignin exhibited a higher yield (70.70%), which can be attributed to its elevated ash content.

**Figure 3:**
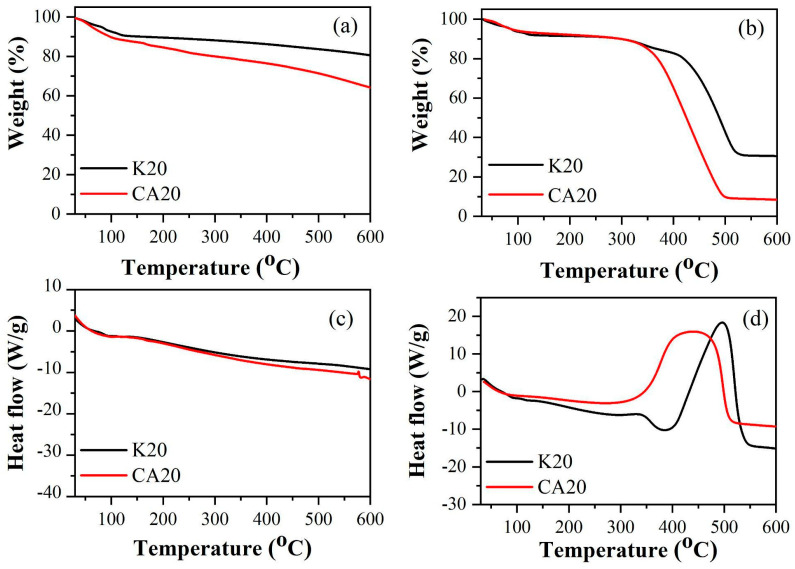
Thermogravimetric curves of biochar in nitrogen (**a**) and air (**b**) atmospheres and DSC curves in nitrogen (**c**) and air (**d**).

Figure 3 exhibit the thermogravimetric (TG) and differential scanning calorimetry (DSC) curves of both biochars under nitrogen and air atmospheres.

In the DSC curves of the biochars, under a nitrogen atmosphere, a continuous endothermic process was observed throughout the thermal analyses starting at approximately 100 °C. Under a synthetic air atmosphere, the DSC curve of CA20 exhibited not only continuous endothermic events during the combustion of the material but also an exothermic peak at approximately 430 °C. Additionally, in synthetic air, a prominent exothermic peak was observed at around 495 °C for K20.

The thermal profiles were employed to perform proximate analysis calculations, following the methodology described in [25]. Table 1 shows the loss percentage in weight of biochars as a function of temperature and atmosphere used. The peak temperatures of the DTG (Tmax) are presented in Table 1.

The biochar derived from sugarcane bagasse lignin exhibited an ash content below 10%. A comparable value of approximately 13% was reported for lignin-derived biochar in a previous pyrolysis study [34], while lignin biochars have generally shown ash contents ranging from 5% to 15% [35].

The elevated ash content observed in the kraft lignin biochar (K20, 30.60%), suggested the presence of salts, which was confirmed in subsequent analyses. This result is consistent with the kraft pulping process, which employs sodium hydroxide and sodium sulfide solutions for lignin extraction [36]. The high ash content of K20 led to a lower volatile matter fraction (11.70 wt%) compared with the biochar obtained from sugarcane bagasse lignin (CA20, 25.10 wt%).

Moisture contents below 10% are generally desirable, as higher values can decrease the energy density of biomass-derived materials [37]. The moisture contents of K20 and CA20 were 6.60% and 6.00%, respectively.

Both biochars presented experimental fixed carbon contents exceeding 50%, with the sugarcane bagasse lignin biochar exhibiting the highest value. Similar findings were reported by [38], who observed approximately 58% fixed carbon content for kraft lignin biochar. The fixed carbon content of lignin biochar, obtained by [35] was above 60%, observing, as expected, an increase in the percentage of fixed carbon, with the increase in the pyrolysis temperature.

The lignin source and extraction method played decisive roles in conferring favorable characteristics to the sugarcane bagasse lignin biochar, as evidenced by the proximate analysis results, which were further supported by the higher heating value (HHV) measurements.

In this context, the HHVs of kraft lignin and sugarcane bagasse lignin were 22.36 MJ·kg^−1^ and 22.78 MJ·kg^−1^, respectively. According to [39] lignin typically exhibits HHVs ranging between 22.20 MJ·kg^−1^ and 28.50 MJ·kg^−1^. The sugarcane bagasse lignin biochar (CA20) presented a higher specific energy content (HHV = 25.95 MJ·kg^−1^) compared with kraft lignin biochar (K20, HHV = 23.05 MJ·kg^−1^), highlighting its greater potential as a solid biofuel.

The results of the proximate analyses and higher heating value determinations for both biochars are summarized in Table 2.

### 3.3. Thermal Profile of Biochars

**Figure 4:**
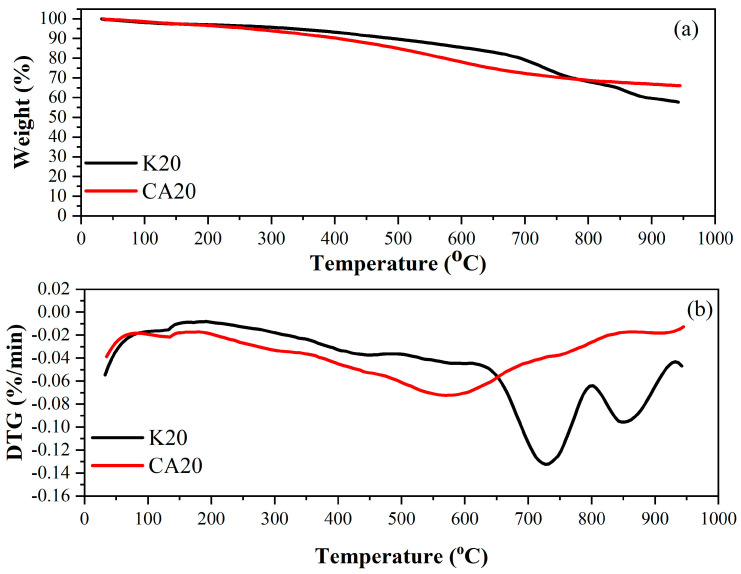
Thermogravimetric (**a**) and DTG curves (**b**) of sugarcane bagasse lignin biochar and kraft lignin biochar under a nitrogen atmosphere.

Figure 4 shows the thermogravimetric and DTG curves of sugarcane bagasse lignin biochar (CA20) and kraft lignin biochar (K20) under a nitrogen atmosphere. These analyses provided an extended thermal profile of both materials, employing the same heating rate used in the initial thermal analysis of sugarcane bagasse lignin and during the pyrolysis experiments (20 °C min^−1^).

The thermal behavior of the commercial kraft lignin biochar revealed a more pronounced mass loss at higher temperatures. From ambient temperature up to 150 °C, a weight loss of approximately 2.46% was observed, primarily due to moisture evaporation. Between 150 °C and 500 °C, the thermogravimetric curve of K20 showed a modest mass loss of 7.80%, followed by a more substantial loss of 32.20% in the 500–950 °C range. At the final temperature, 57.54% of K20 biochar remained as residual mass. For the K20 DTG profile, the peak decomposition temperature (Tmax) was approximately 730 °C

In contrast, the sugarcane bagasse lignin biochar (CA20) exhibited a higher mass loss at lower temperatures compared with K20. Between ambient temperature and 150 °C, CA20 exhibited a 2.40% mass loss was recorded, corresponding to moisture release. From 150 °C to 500 °C, CA20 presented a 12.94% mass loss, and an additional 18.60% loss occurred between 500 °C and 950 °C. At the end of the analysis, the residual mass of CA20 under a nitrogen atmosphere was 66.06%.

For the CA20 DTG profile, the peak decomposition temperature was approximately 570 °C.

Table 3 presents the mass loss of the biochars under a nitrogen atmosphere.

### 3.4. Elementary Analysis of Materials

The elemental compositions of both lignins are summarized in Table 4. The results for kraft lignin and sugarcane bagasse lignin were consistent with those reported in the literature, with elemental percentages varying according to the extraction method employed. Notably, sulfur was detected in kraft lignin but was absent in both the sugarcane bagasse lignin and its corresponding biochar, reflecting the distinct delignification processes used [7,40].

The relative increase in carbon content observed in the sugarcane bagasse lignin biochar suggests enhanced hydrophobicity, a reduction in hydrogen content, and oxygen depletion resulting from the slow pyrolysis treatment. This compositional shift indicates a higher degree of carbonization, which contributes to the improved stability and potential functional performance of the biochar.

The oxygen content calculated for the kraft lignin and sugarcane bagasse biochars was 11.21 wt% and 26.37 wt%, respectively, while the O/C ratios obtained for these biochars were 0.15 and 0.32, respectively. The lower O/C ratio of the kraft lignin biochar reflects its higher ash content compared to the biochar derived from sugarcane bagasse lignin.

### 3.5. X-Ray Diffraction (XRD) of Lignins and Biochar

**Figure 5:**
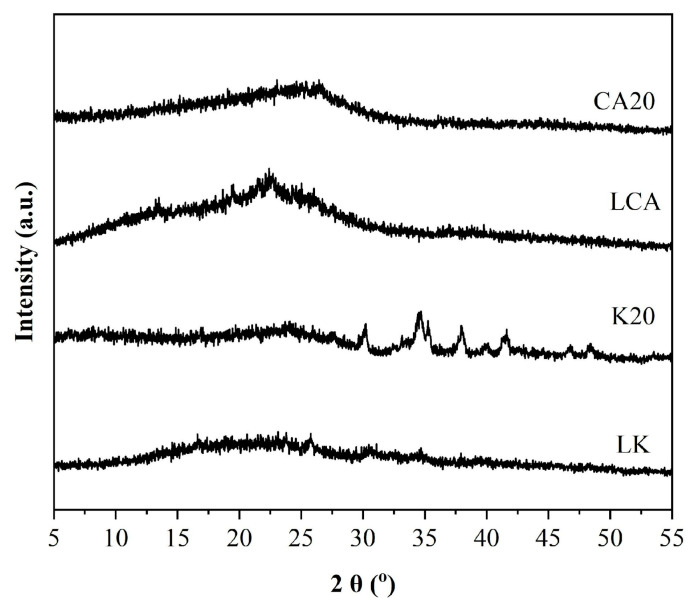
X-Ray diffraction pattern of lignins and their biochars.

The X-Ray diffraction (XRD) patterns of lignin extracted from sugarcane bagasse (LCA) and its corresponding biochar (CA20) indicate that both materials are amorphous, as shown in Figure 5.

The same figure also displays the XRD patterns of kraft lignin (LK) and its biochar (K20). The diffractogram of K20 reveals low structural ordering and reveals the contaminant phase, identified as sodium carbonate (Na_2_CO_3_). This phase became more prominent due to the relative concentration increase after pyrolysis. Similar crystalline salt phases, particularly Na_2_CO_3_ on the surface of carbonaceous materials derived from black liquor, were also reported by [41].

### 3.6. Characterization by Fourier Transform Infrared Spectroscopy of Lignins and Biochar

**Figure 6:**
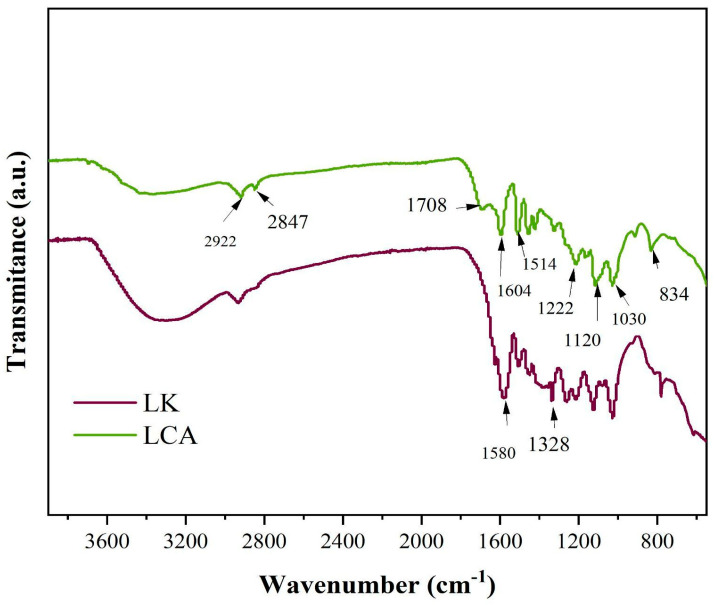
Infrared spectra of kraft lignin (LK) and sugarcane bagasse lignin (LCA).

Figure 6 shows selected absorption bands in the infrared spectra of the lignin samples. For both kraft lignin (LK) and bagasse-derived lignin (LCA), prominent bands are observed at approximately 1605 cm^−1^ and 1515 cm^−1^, characteristic of aromatic ring vibrations in the phenylpropane units of lignin. A distinct band at ~ 1420 cm^−1^, is also present, attributed to aromatic skeletal vibrations coupled with in-plane C–H deformation [42,43,44].

The band at 2922 cm^−1^ corresponds to C–H stretching, vibrations of methyl and/or methylene groups. A band near 1330 cm^−1^ is characteristic of syringyl (S) units, resulting from C–O stretching in sinapyl alcohol. Additionally, the ~1120 cm^−1^, band is assigned to in-plane aromatic C–H deformation. For guaiacyl (G) units, derived from coniferyl alcohol, band near 1220 cm^−1^ and 1030 cm^−1^ are observed, corresponding to C–C, C–O, and C=O stretching, and in-plane C–H deformation, respectively [42,43,44].

Other absorption features include a band at 2847 cm^−1^ (common to both lignins), attributed to aliphatic methylene C–H stretching, and a band at 1708 cm^−1^ in LCA, assigned to conjugated carbonyl/carboxyl stretching [45]. A peak at ~ 834 cm^−1^, observed in LCA, corresponds to out-of-plane C–H vibrations at positions is attributed to out-of-plane C–H vibrations at positions 2 and 6 of S units and at all positions of H units, derived from p-coumaryl alcohol [46].

Furthermore, a broad band centered at 1030 cm^−1^ in LCA is attributed to C–OH stretching in G units [47]. A medium-intensity band at 1501 cm^−1^ is associated with C–H deformation in methyl and methylene groups. A shoulder at approximately 1294 cm^−1^ is linked to C–O stretching in syringyl units.

**Figure 7:**
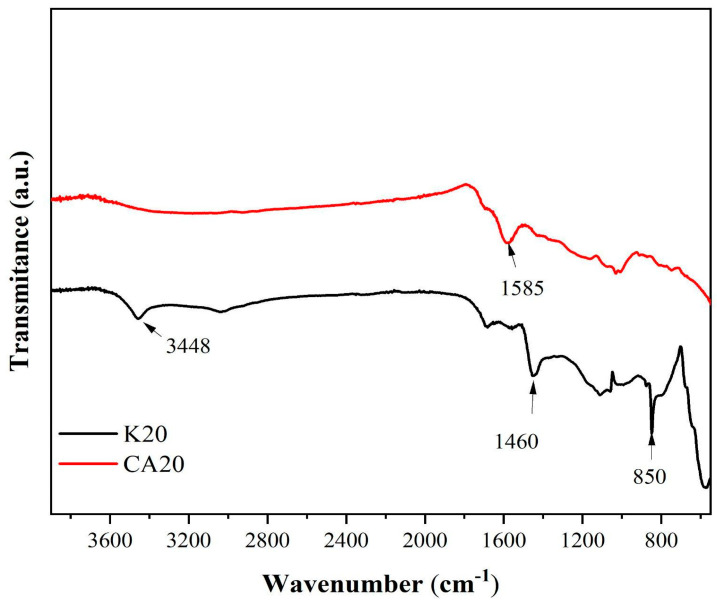
Infrared spectra of the biochars derived from kraft lignin (K20) and sugarcane bagasse lignin (CA20).

Figure 7 presents the infrared spectra of the biochars derived from kraft lignin (K20) and sugarcane bagasse lignin (CA20).

In the spectrum of K20, several bands are identified, including a broad band at 3448 cm^−1^ corresponding to O–H stretching vibrations, a band at 1460 cm^−1^ associated with C–H deformation and aromatic ring vibrations, and a band at 850 cm^−1^ attributed to asymmetric in-plane bending of isolated aryl C–H groups [48].

For CA20, a distinct band at 1585 cm^−1^ is observed, which is attributed to the presence of carbonyl and carboxyl functional groups.

### 3.7. Analysis by Raman Spectroscopy

**Figure 8:**
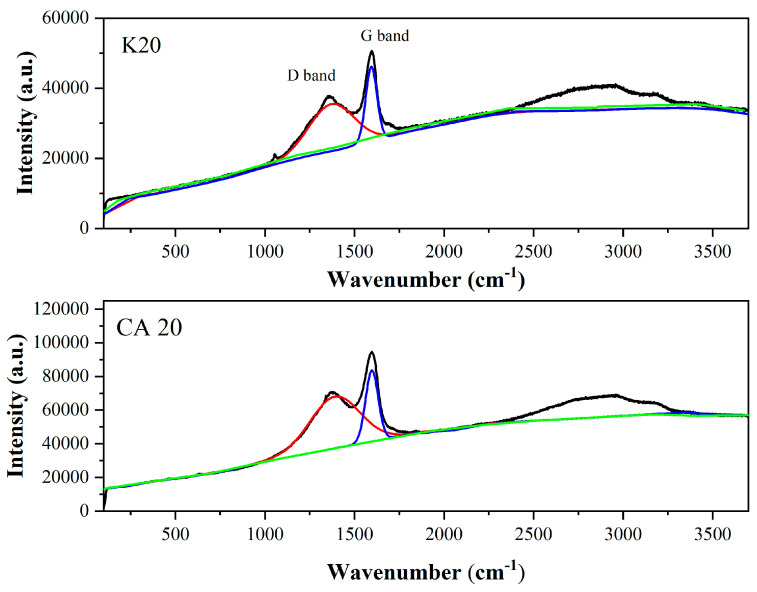
Raman spectra of lignin biochars. In red the integration area in the D band, in blue the integration area in the G band. In green base line.

The analysis of the D and G bands, depicted in Figure 8, provides insights into structural disorder and defects in biochar samples. The D band, centered at ~1360 cm^−1^, is associated with structural defects in sp^2^-hybridized carbon and indicates the presence of amorphous carbon domains [49]. In contrast, the G band, located at ~1590 cm^−1^, corresponds to the in-plane stretching of sp^2^-hybridized carbon atoms and is characteristic of graphitic (crystalline) structures [50].

The presence of both D and G bands suggests a mixed structure comprising amorphous and crystalline carbon [49]. The ratio was employed to assess the structural properties of the biochar.

It is noteworthy that Raman spectroscopy and X-Ray diffraction (XRD) analyses provided complementary structural information. Raman analysis revealed a highly disordered carbon structure with small graphitic domains, while XRD confirmed the predominantly amorphous nature of the materials. These results indicate that both short-range (Raman) and long-range (XRD) ordering are limited in K20 and CA20.

For the K20 biochar, the integration area in the D band was in the order of 9.4974 × 10^19^, while in the G band, the integration area was in the order of 9.2423 × 10^19^. For the CA20 biochar, the integration area in the D band was in the order of 1.6226 × 10^20^, whereas in the G band it was in the order of 1.5508 × 10^20^.

In this study, the intensity ratio (ID/IG) for kraft lignin biochar (K20) and sugarcane bagasse lignin biochar (CA20) was, respectively, 1.028 and 1.089, indicating a degree of disorder similar to that reported in [41].

The drift coefficient (α) for biochars calculated from the intensity of points A at 800 cm^−1^ (IAr), B at 1800 cm^−1^ (IBr), and the intensity of the D band (IDr), was greater than 0.48.

The biochar yield (CY) was greater for K20. A nearly linear correlation between biochar yield and residual volatile content (RV) has also been reported [27]; the RV value for biochars was greater than 20.00%.

Using this methodology, the ash-free dry volatile content (Vdaf) for sugarcane bagasse lignin biochar and kraft lignin biochar was determined to be, respectively, 50.13 wt% and 54.64 wt%.

The H/C ratio decreases linearly with decreasing Vdaf, as described in [27]. Biochars with volatile matter < 80% and an O/Corg ratio > 0.2, H/Corg > 0.4, such as lignin biochars, indicate a moderate potential for carbon sequestration [51].

In this study, the hydrogen-to-carbon (H/C) ratio of the biochars, as determined from the α coefficient obtained via Raman spectroscopy, was considerably higher than the H/C ratio measured through elemental analysis. The primary challenge in accurately determining the H/C ratio lies in the inherent properties of hydrogen, which is a light and volatile element, thereby complicating its precise quantification. Nevertheless, both analytical techniques revealed H/C ratios greater than 0.5 for all biochar samples. Furthermore, in both methods of analysis, the H/C ratio was higher for the kraft lignin-derived biochar compared to the sugarcane bagasse lignin-derived biochar.

According to the Van Krevelen diagram for biochars, lower contents of oxygen and hydrogen are preferred—particularly when targeting applications such as solid fuels or decarbonizing agents. This trend was observed in the lignin-derived biochar from sugarcane bagasse, which showed an O/C ratio of 0.34. This corresponds to an O/C ratio closely matching the value obtained via elemental analysis (0.32).

The H/Corg and O/Corg ratios in biochars are recommended by the European Biochar Foundation and the International Biochar Initiative as indicators for assessing biochar stability. Thus, a decreasing H/C ratio in biochars indicates superior aromatic condensation and increased biochar stability, while a decreasing O/C and H/C ratios indicate increased hydrophobicity and aromaticity in biochars [52].

Typically, a high H/C ratio (>1.0) indicates a higher proportion of aliphatic bonds and low carbonization, resulting in more volatile compounds and lower energy density. Conversely, a low O/C ratio (<0.2) is indicative of stable, hydrophobic materials with higher energy content. In contrast, an O/C ratio >0.5 suggests high oxygen content and low calorific value [53,54].

Broad-range Raman analysis (150–3500 cm^−1^) also revealed signals potentially corresponding to overtone and combination bands, such as 2D and 2G, commonly observed in graphitic materials. Polycyclic aromatic hydrocarbons present in K20 and CA20 may have contributed to combination bands such as G + D and G + A. Furthermore, the use of a 514 nm laser likely facilitated the detection of these overtones [55].

Table 5 presents the parameters obtained using the Raman spectroscopy technique.

### 3.8. Scanning Electron Microscopy Coupled with Energy Dispersive Spectroscopy

**Figure 9:**
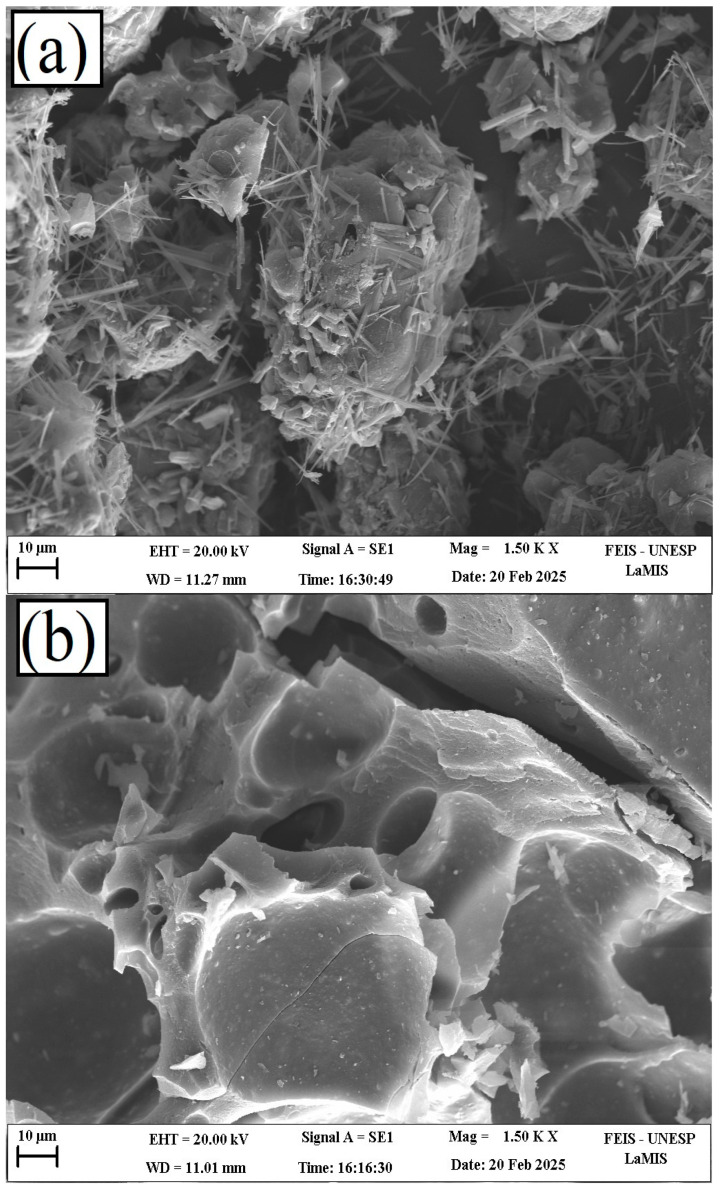
Micrographs of kraft lignin biochar (**a**) and sugarcane bagasse lignin biochar (**b**).

Figure 9 presents scanning electron microscopy (SEM) images of the biochars derived from sugarcane bagasse lignin (CA20) and kraft lignin (K20), highlighting significant variations in particle size and morphology. These differences are influenced by the milling process, which can cause fragmentation, as well as by the potential deposition of volatile matter forming granular surface structures [34].

The SEM images of CA20 predominantly show smooth, flat, and compact surfaces. This morphology is attributed to the intrinsic characteristics of sugarcane bagasse lignin and the behavior of lignin during pyrolysis, which involves particle fusion and aggregation under thermal decomposition [56]. Depressions observed on the surfaces likely result from volatile release and structural rearrangements occurring during carbonization.

In contrast, K20 exhibits more irregular surface features, including filamentous structures and heterogeneous topographies. While some compact regions are present, the overall morphology is rougher and less uniform compared to CA20, likely due to differences in the structural composition and thermal behavior of kraft lignin during pyrolysis.

Table 6 presents the results of the elemental analysis performed via scanning electron microscopy (SEM) coupled with energy-dispersive X-Ray spectroscopy (EDS) on the biochar surfaces.

The biochar derived from sugarcane bagasse lignin exhibited a silicon content of 2.01 wt%, consistent with values typically reported for biochars produced from agro-industrial residues [7,57]. Trace elements such as aluminum (1.53 wt%) and iron (0.88 wt%) were also detected. The major elements present were carbon and oxygen, with CA20 showing the highest surface carbon content among the samples analyzed (67.59 wt%), which is indicative of a higher energy density.

In contrast, kraft lignin biochar (K20) contained elevated levels of sulfur (0.91 wt%), nitrogen (4.15 wt%), and sodium (11.21 wt%). These elements are characteristic of kraft lignin due to the use of sodium sulfide during the alkaline pulping process, which typically results in sulfur concentrations ranging from 1.5 to 3.0 wt% [58]. The presence of these elements contributes to a reduction in energy density and potential limitations for some applications. Additionally, potassium (0.53 wt%) and silicon (0.01 wt%) were detected in K20.

### 3.9. True Density Analysis

The true densities of kraft lignin and sugarcane bagasse lignin, determined by helium pycnometry, were 1.2939 g/cm^3^ and 1.2911 g/cm^3^, respectively. These values are close to the lower end of the typical range for lignin at room temperature (1.3000–1.5000 g/cm^3^) [59], indicating consistency with previously reported data.

Following pyrolysis, the true densities increased to 1.5067 g/cm^3^ for kraft lignin biochar (K20) and 1.3881 g/cm^3^ for sugarcane bagasse lignin biochar (CA20). This increase is attributed to the thermal degradation and structural rearrangement of lignin during pyrolysis. However, both biochars exhibited true densities significantly lower than that of graphite (2.2500 g/cm^3^) [60].

Typically, the inorganic component of a biochar possesses a greater density than its organic counterpart. Consequently, in biochars with elevated ash content, the experimentally determined true densities may be moderately overestimated [61].

According to [62], increased true density in biochars may correlate with higher porosity. Additionally, [60] reported that raising the pyrolysis temperature increases the true density of sugarcane bagasse biochar. In that study, a feedstock with slightly higher initial density (1.3100 g/cm^3^) produced a biochar with a true density of 1.5900 g/cm^3^ after pyrolysis at 500 °C, exceeding the values observed for lignin-derived biochars in the present work.

## 4. Conclusions

Lignin is an undervalued byproduct of the second-generation ethanol industry. Its conversion via slow pyrolysis yields a solid carbon-rich material, known as biochar, offering a promising route for lignin valorization. Biochar represents a promising route for adding value to lignin. When applied as a solid fuel or precursor for carbon-based materials, sugarcane bagasse lignin biochar demonstrates viability as a sustainable alternative.

This research employed purified lignin derived from sugarcane bagasse as the primary substrate for biochar synthesis instead of using the untreated bagasse. Compared to commercial kraft lignin and its biochar, both the sugarcane bagasse lignin and its corresponding biochar exhibited higher carbon content, reflecting the influence of extraction methods.

FTIR analysis confirmed the presence of characteristic lignin absorption bands at ~1605 cm^−1^ and ~1515 cm^−1^ in both lignins. However, kraft lignin and its biochar contained elevated levels of sodium—elements absent in sugarcane bagasse lignin. XRD analyses confirmed the amorphous nature of the sugarcane biochar and revealed the presence of sodium carbonate in kraft lignin biochar.

The lower oxygen content observed in the sugarcane bagasse lignin biochar suggests superior potential as a solid fuel, as a high O/C ratio is characteristic of biomass with a low calorific value. An O/C ratio below 0.5 in biochars is indicative of low oxygen content, which enhances the energy density of the biochar compared to the original biomass.

The elemental composition of solid fuels is critical for understanding their performance as solid biofuels. Sulfur—present only in the kraft lignin and its corresponding biochar—can contribute to energy release during combustion; however, it also promotes the formation of toxic gases. In this context, lignin-derived biochar from sugarcane bagasse has demonstrated a lower contaminant content.

The pyrolysis temperature employed in this study resulted in a high fixed carbon yield in the sugarcane bagasse lignin biochar (exceeding 60%). The elevated carbon content directly influences the higher calorific value, as carbon oxidation serves as the primary source of energy during combustion. Accordingly, the sugarcane bagasse lignin biochar exhibited high energy density combined with low ash content.

Elemental analysis and Raman spectroscopy revealed hydrogen-to-carbon (H/C) ratios greater than 0.5 for all biochar samples. The reduction in the H/C ratio in biochars will be explored in future studies to enhance the material’s stability, considering that only a moderate degree of carbonization was achieved in the present study, and the sugarcane bagasse lignin-derived biochar exhibited moderate stability.

One specific area requiring further improvement in future studies is the characterization of the textural properties of the materials, in order to further enhance their overall performance and broaden their potential applications when compared to biochars derived from other lignin-rich biomasses.

## Figures and Tables

**Table 1:** Mass loss of biochars under nitrogen and synthetic air atmospheres.

Mass Loss in Synthetic Air (wt%)					Mass Loss in Nitrogen (wt%)	
Biochar	30–105 °C Range	105–600 °C Range	30–600 °C Range	Tmax (°C)	105–600 °C Range	Tmax (°C)
K20	6.60	62.80	69.40	496.40	11.70	86.59
CA20	6.00	85.60	91.60	430.68	25.10	56.24

**Table 2:** Proximate analysis and higher heating value of biochars.

	K20 (wt%)	CA 20 (wt%)
Proximate analysis		
Moisture content	6.60	6.00
Ash content	30.60	8.40
Fixed carbon	51.10	60.50
Volatile matter	11.70	25.10
Higher Heating Value (MJ/kg)		
	23.05	25.95

**Table 3:** Mass loss of the biochars under a nitrogen atmosphere.

Mass Loss Under A Nitrogen Atmosphere (wt%)				
Biochar	30–150 °C Range	150–500 °C Range	500–950 °C Range	Tmax (°C)
K20	2.46	7.80	32.20	730
CA20	2.40	12.94	18.60	570

**Table 4:** Elemental Analysis of the Materials.

	Elements (wt%)				
Materials	C	H	S	N	H/C
LK	49.20	4.71	1.96	0	1.14
LCA	54.29	4.89	0	0.73	1.07
K20	54.77	2.66	0.76	0	0.58
CA 20	61.64	2.74	0	0.85	0.53

**Table 5:** Parameters obtained by Raman.

Parameter	K20	CA20
ID/IG	1.028	1.089
α	0.60	0.48
CY (%)	44.64	41.14
RV (%)	24.39	20.62
Vdaf (%)	54.64	50.13
H/C	0.95	0.87
O/C	0.38	0.34

**Table 6:** Elemental analysis of biochar.

Element	K20 (wt%)	CA20 (wt%)
C	44.86	67.59
O	38.33	27.98
Si	0.01	2.01
S	0.91	-
Na	11.21	-
N	4.15	-
K	0.53	-
Fe	-	0.88
Al	-	1.53

## Data Availability

The original contributions presented in this study are included in the article. Further inquiries can be directed to the corresponding authors.

## Abbreviations

The following abbreviations are used in this manuscript:

LCA	Sugarcane bagasse lignin
LK	Kraft lignin
CA20	Sugarcane bagasse lignin biochar
K20	Kraft lignin biochar
E2G	Second-generation ethanol
